# Cytological Screening for Pre-Invasive Cervical Cancer

**Published:** 1965-04

**Authors:** J. S. Cornes, Valerie Way, M. B. Wingate

**Affiliations:** United Bristol Hospitals; United Bristol Hospitals; United Bristol Hospitals


### CYTOLOGICAL SCREENING FOR PRE-INVASIVE CERVICAL CANCER: A PILOT STUDY IN BRISTOL

BY

J. S. CORNES, VALERIE WAY, AND M. B. WINGATE

*United Bristol Hospitals*

A study of the mortality figures from the annual report of the Registrar General shows that approximately one woman in every seventy will develop cancer of the cervix and one in every hundred will die from the disease. In 1910 Rubin described a condition, since called carcinoma in situ or pre-invasive cancer, in which malignant cells were present on the surface of the cervix without showing any signs of invasion. In 1944 Te Linde and Galvin noted a relation between carcinoma in situ and early invasive cancer, and, in an attempt to find and to treat it, the method of cytological examination of exfoliated cervical cells was developed. McLaren (1963) has stated that if cervical cancer can be diagnosed at its pre-invasive stage the cure rate, or more accurately the prevention rate, is 100 per cent.

Carcinoma in situ is usually symptomless and the cervix is normal in appearance or may present an apparently benign lesion. For this reason the practice of screening "well women" in the age group at risk is now becoming established in many countries. Few total population studies have been reported but the incidence of "pick up" in these varies between three and five per thousand. If screening is confined to patients who attend gynaecological or obstetric clinics the incidence may be as high as 11 per thousand (S.E. Scotland College of General Practitioners, 1958).

The average age of women with carcinoma in situ is 38 years but the peak incidence occurs in the 30 to 35 year age group. As the average age of women with invasive carcinoma is 48 years there appears to be an average delay of ten years between the development of in situ and invasive cancer. Over 95 per cent of these women are married with two or more children and just under half have had sexual intercourse before the age of 20 years.

### DIAGNOSIS

The consensus of opinion is that a smear taken by means of a spatula, such as that designed by Ayre, provides the most representative material from the cervix. There is, however, no need to use this relatively expensive spatula, a simple wooden one is equally effective if correctly applied.

The cervix is exposed with a speculum, the spatula applied gently but firmly against the external os, and rotated round the whole 360° of the ectocervix. The sample of epithelium obtained is then quickly spread onto two clean microscope slides marked with the patient's name. These are immediately put into 95 per cent alcohol in a plastic slide container fitted with a screw top. It is important not to allow the smear to dry before fixing in alcohol. The smears may be left in 95 per cent alcohol for up to two weeks. After fixation the smears are stained with Papanicolaou's stains.

For the purpose of reporting cervical smears are classified into five separate grades or categories:

Grade I, normal smear.

Grade II, infective smear.

Grade III, dyskaryotic cells present.

IIIA, superficial dyskaryosis.

IIIB, basal cell dyskaryosis.

Grade IV, differentiated malignant cells present.

Grade V, undifferentiated malignant cells present.

The earliest malignant cellular change now recognized is termed dyskaryosis, and a distinction is drawn between superficial and basal cell dyskaryosis. The affected cells have normal cytoplasm but abnormal nuclei, the nuclear chromatin being irregular in distribution and exhibiting clumping. Dyskaryosis is a reversible phenomenon and in women so affected less than half eventually develop cervical cancer (Table I).

TABLE I

Cytological follow-up study on patients with dyskaryotic cells in cervical smears (after Attwood)

| Type of dyskaryosis | Cytological follow-up: approximate percentage showing— | | |
|---|---|---|---|
| | regression | persistent dyskaryosis | positive smear |
| Superficial | 68 per cent | 14 per cent | 18 per cent |
| Basal | 33 per cent | 12 per cent | 55 per cent |

Three types of differentiated squamous carcinoma cell are described in positive smears: fibre shaped, tadpole shaped, and third type differentiated. Third type differentiated cells are the ones most commonly found. They are round or oval in shape, contain a small amount of dense cytoplasm, and have centrally placed malignant looking nuclei (Plate VI). If these are the only type of cell present in the smear the diagnosis is likely to be carcinoma in situ with or without micro-invasion of the underlying stroma. The smear from a frankly invasive carcinoma usually has both differentiated and undifferentiated cells, the latter having virtually no cytoplasm and grossly abnormal nuclei.

The presence of suspicious or frankly malignant cells in a cervical smear does not make a diagnosis of carcinoma in situ certain. To confirm the diagnosis an adequate cone biopsy of the cervix must be performed, and the pathologist must examine sections from many parts of the cone to exclude invasive or micro-invasive carcinoma (Plate VII).

Cone biopsy is frequently curative, and in younger women all that is subsequently required is regular follow up examination and cytological screening. The operation does not increase the risk of infertility, abortion or stillbirth, and does not give rise to difficulty with subsequent vaginal delivery.

### PILOT STUDY

As part of a research project routine cervical smears were taken from all patients attending gynaecological out-patients in the Professorial Unit over a period of eight months. Smears were also taken for two months from patients booking for delivery at one of the antenatal clinics.

In all, 862 patients were examined, 833 from gynaecological clinics, and 29 from the antenatal clinic. Malignant cells were found in the smears from 22 patients, and dyskaryotic cells in a further 17 patients (Table II).

TABLE II

Results of Survey

| Cytological diagnosis | Number of Patients | Age range | Number confirmed by biopsy | Number awaiting biopsy | Incorrect cytological diagnosis |
|---|---|---|---|---|---|
| Invasive carcinoma | 5 | 37–66 | 4 | 1 | — |
| Carcinoma in situ | 5 | 25–58 | 3 | 2 | — |
| Adeno-carcinoma | 12 | 51–80 | 7 | 3 | 2 |
| Basal dyskaryosis | 12 | 28–58 | 2 | 9 | 1 |
| Superficial dyskaryosis | 5 | 22–35 | — | — | — |

Only two of the ten patients with invasive or in situ carcinoma had clinically suspicious cervices, the other eight were described as benign in appearance. One of the five patients with in situ carcinoma was found in the antenatal clinic. Confirmative biopsy will be obtained shortly after delivery. Examination of the cone biopsies from two of the patients with carcinoma in situ showed small areas of micro-invasion.

Although this pilot study was not primarily concerned with the detection of carcinoma of the body of the uterus no less than twelve patients had exfoliated malignant endometrial cells in their cervical smears. All these patients were referred with menorrhagia or post-menopausal bleeding, and two had been placed on the routine admission list because of their symptoms and signs (Plates VIII and IX). The cytological diagnosis of carcinoma of the body was incorrect on two occasions when large atypical glandular cells were found in the cervical smears. Examination of the endometrium showed the diagnosis to be endometrial hyperplasia without evidence of malignant change.

### DISCUSSION

The rate of "pick up" of malignant cells in the cervical smears examined is in the region of twice the number which could have been expected when compared with other surveys. This may in part be explained by the selection of patients, in that they attended a gynaecological or obstetric clinic, and in part to the recognized fact that screening for the first time in any population provides an initially high positive result.

It was difficult at first to be certain of the emphasis to be placed on the finding of dyskaryotic cells in cervical smears. Their presence, however, together with malignant cells in many of the positive smears, has led to an increasingly serious view being taken of basal cell dyskaryosis. It is now the practice to have the smear repeated in three months, and if it is still positive it is felt necessary to examine a cone biopsy of the cervix. Three patients with basal dyskaryosis were so treated. Two showed typical dysplasia and one a cervical erosion with micro-polypi and severe chronic cervicitis. No biopsy was taken from a patient with superficial cell dyskaryosis.

It was found that careful handling of the cone biopsy material was of great importance. The cone must be pinned out and fixed before sectioning, or the squamo-columnar junction may be lost, and unless parallel blocks are taken across the whole specimen small areas of invasion or micro-invasion are easily overlooked. Since cervical cancer commonly begins at the squamo-columnar junction sections which fail to show it must be regarded as unsatisfactory and the biopsy reported as inconclusive.

### CONCLUSIONS

This pilot study has produced results which, in our opinion, justify routine cytological screening of the population at risk for the early detection of cancer of the cervix. It was distressing to have to refuse requests from individual doctors and from organizations to screen women who had made personal requests to them.

Although the cervical smear is not the most effective means of providing material for the cytological detection of cancer of the body of the uterus it was of interest to note the number of positive smears obtained in patients with this condition.

The study has borne out the contention that pre-invasive cervical cancer cannot be detected by clinical examination alone, and that the same observation applies to some cases of early invasive cancer.

### ACKNOWLEDGEMENTS

We wish to express our gratitude to the Medical Research Committee of the United Bristol Hospitals for financing this pilot study. J. S. C. and V. M. are indebted to the Board of Governors of the United Bristol Hospitals, and to Prof. H. C. McLaren and Miss M. E. Attwood of the Dept. of Obstetrics and Gynaecology of Birmingham University, for training in cytology.

### REFERENCES

Attwood, M. E. (1964). Personal communication.

McLaren, H. C. (1963). "The Prevention of Cervical Cancer". English University Press Ltd., London.

Rubin, I. C. (1910). *Amer. J. Obstet.*, **62,** 668.

S.E. Scotland Faculty of College of General Practitioners (1958). *Lancet*, **ii,** 845.

Te Linde, R. W. and Galvin, G. A. (1944). *Amer. J. Obstet. Gynec.*, **48,** 774.

Wingate, M. B. (1963). Prospective Study on Carcinoma in Situ. Royal College of Obstetricians and Gynaecologists.

